# Multidimensional Role of Silicon to Activate Resilient Plant Growth and to Mitigate Abiotic Stress

**DOI:** 10.3389/fpls.2022.819658

**Published:** 2022-03-23

**Authors:** Rakeeb Ahmad Mir, Basharat Ahmad Bhat, Henan Yousuf, Sheikh Tajamul Islam, Ali Raza, Masood Ahmad Rizvi, Sidra Charagh, Mohammed Albaqami, Parvaze A. Sofi, Sajad Majeed Zargar

**Affiliations:** ^1^Department of Biotechnology, School of Life Sciences, Central University of Kashmir, Ganderbal, India; ^2^Centre of Research for Development, University of Kashmir, Srinagar, India; ^3^Department of Biotechnology, School of Biosciences and Biotechnology, Baba Ghulam Shah Badshah University, Rajouri, India; ^4^Key Laboratory of Ministry of Education for Genetics, Breeding and Multiple Utilization of Crops, Oil Crops Research Institute, Center of Legume Crop Genetics and Systems Biology/College of Agriculture, Fujian Agriculture and Forestry University (FAFU), Fuzhou, China; ^5^Department of Chemistry, University of Kashmir, Srinagar, India; ^6^State Key Laboratory of Rice Biology, China National Rice Research Institute, Chinese Academy of Agricultural Science, Hangzhou, China; ^7^Department of Biology, Faculty of Applied Science, Umm Al-Qura University, Makkah, Saudi Arabia; ^8^Division of Genetics and Plant Breeding, Sher-e-Kashmir University of Agricultural Sciences and Technology of Kashmir, Srinagar, India; ^9^Proteomics Laboratory, Division of Plant Biotechnology, Sher-e-Kashmir University of Agricultural Sciences and Technology of Kashmir (SKUAST-K), Srinagar, India

**Keywords:** silicon, phytohormones, abiotic stress, sustainable agriculture, climate change, non-essential elements

## Abstract

Sustainable agricultural production is critically antagonistic by fluctuating unfavorable environmental conditions. The introduction of mineral elements emerged as the most exciting and magical aspect, apart from the novel intervention of traditional and applied strategies to defend the abiotic stress conditions. The silicon (Si) has ameliorating impacts by regulating diverse functionalities on enhancing the growth and development of crop plants. Si is categorized as a non-essential element since crop plants accumulate less during normal environmental conditions. Studies on the application of Si in plants highlight the beneficial role of Si during extreme stressful conditions through modulation of several metabolites during abiotic stress conditions. Phytohormones are primary plant metabolites positively regulated by Si during abiotic stress conditions. Phytohormones play a pivotal role in crop plants’ broad-spectrum biochemical and physiological aspects during normal and extreme environmental conditions. Frontline phytohormones include auxin, cytokinin, ethylene, gibberellin, salicylic acid, abscisic acid, brassinosteroids, and jasmonic acid. These phytohormones are internally correlated with Si in regulating abiotic stress tolerance mechanisms. This review explores insights into the role of Si in enhancing the phytohormone metabolism and its role in maintaining the physiological and biochemical well-being of crop plants during diverse abiotic stresses. Moreover, in-depth information about Si’s pivotal role in inducing abiotic stress tolerance in crop plants through metabolic and molecular modulations is elaborated. Furthermore, the potential of various high throughput technologies has also been discussed in improving Si-induced multiple stress tolerance. In addition, a special emphasis is engrossed in the role of Si in achieving sustainable agricultural growth and global food security.

## Introduction

The reduction in crop productivity by 51–82% on a global scale is primarily influenced by abiotic stress factors, such as drought, salinity, heat, cold, toxic heavy metals, hypoxia and anoxia, waterlogging, and nutrient imbalance (Cooke and Leishman, 2016; Zahra et al., 2021; Raza et al., 2022). Contemporary climate change notably imparts drought and heat stress, two major abiotic stress factors resulting in crop loss and productivity (Zandalinas et al., 2018). For instance, studies report that about a 40% loss in yield in maize (*Zea mays* L.) and wheat (*Triticum aestivum* L.) is primarily due to drought stress (Daryanto et al., 2016). Therefore, it is evident that abiotic stress factors are the most severe constraints to global food production, leading to nutritional and food insecurity in the climate-changing era (Del Buono, 2021; Kamenya et al., 2021; Raza, 2022). The last few decades witnessed an increase in the prevalence of abiotic stressors primarily due to unpredictable weather changes induced by global climatic change (Del Buono, 2021; Kamenya et al., 2021; Raza, 2022). Abiotic stressors alter the homeostasis of the physiological, molecular, and biochemical setup of crop plants, such as water relations, nutrient uptake and assimilation, osmotic deregulation, loss of membrane integrity, impairment in enzyme activity, and the most notable decline in photosynthetic efficiency (Moradtalab et al., 2018; Ahanger et al., 2019; Raza, 2021; Raza et al., 2021e). Most of these impairments after exposure to the stress factors are associated with phytohormone production and their transport in underground and aerial parts of plants (Gangwar et al., 2014; Verma et al., 2016; Arif et al., 2021). Moreover, plants primarily respond to these abiotic stressors by producing reactive oxygen species (ROS), which, in turn, induce severe damage to the cellular structures, such as biomembranes and organelles (da Silva and Yücel, 2008; Hasanuzzaman et al., 2020).

To circumvent these harmful effects, plants respond through physiological and molecular mechanistic adjustments to mitigate the adverse impacts of abiotic stressors (Raza, 2021, 2022). Additionally, the defense response to these abiotic stressors includes synthesis of critical proteins linked to metabolism, activation of signal transduction, and regulatory pathways controlled by the expression of a large number of stress-tolerant genes, which, in turn, are regulated by diverse organic and inorganic molecules (da Silva and Yücel, 2008; Hasanuzzaman et al., 2020; Raza et al., 2021e). Among inorganic molecules, Si, formally categorized as the non-essential element, has been recently found to defend against the harmful effects of abiotic stressors in crop plants. The uptake of Si results in the activation of a diverse range of critical genes to mitigate stress conditions to regulate plant growth and development. Si is the second most abundant element found in the earth’s crust (Luyckx et al., 2017; Atta et al., 2019; Zargar et al., 2019). Si is crystalline in structure and forms at least two stable oxide forms, *viz*. Si monoxide (SiO) and silica (SiO_2_). Average soils contain about 28% Si by weight in silicates, alumina-silicates, and Si dioxide, most of which are unavailable to crop plants. Traces of monosilicic acid found in soil are the only form of Si available to plants (Pereira et al., 2013). A later form of Si is soluble in water and is weakly adsorbed by soil. The transport of Si into aerial parts of crop plants occurs through several specific and non-specific transporters. In several crop plants, such as maize, cucumber, rice, and barley; genes, such as *Lsi2*, *Lsi1*, and *Lsi6*, are responsible for uptake of Si in roots and aerial organs (Wang et al., 2015; Ouellette et al., 2017; Ratcliffe et al., 2017). Among these, *Lsi1* and *Lsi6* transporters belonging to aquaporins are abundantly distributed in shoot and root tissues, whereas *Lsi2*, an anion transporter, is found in membranes of root endodermis (Mitani et al., 2011).

**FIGURE 1:**
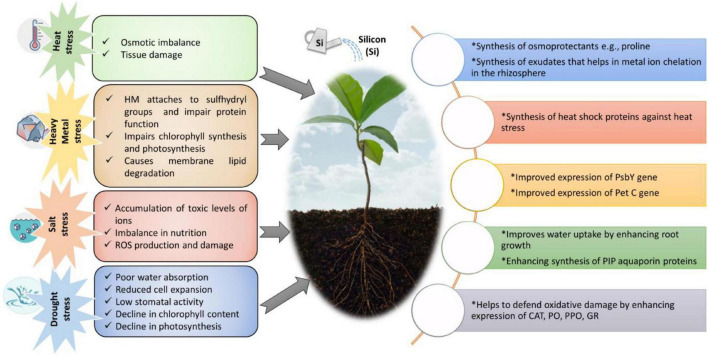
Impact of abiotic stress on various aspects of plant growth and defense mechanisms evoked by application of Si.

Crop plants may grow well in the absence of Si, although in a few plants, such as rice and horsetail, the absence of Si may make them vulnerable to fungal infections (Law and Exley, 2011). Si has been reported to play numerous roles in mitigating abiotic stress conditions (Coskun et al., 2016; Luyckx et al., 2017; Rasoolizadeh et al., 2018; Zhu et al., 2019a; Alamri et al., 2020; Salim et al., 2021). For instance, Si mediates diverse strategies to sequester the metal ions by modulating soil pH, metal speciation, co-precipitation, and compartmentalization (Debona et al., 2017). Recent trends have proved the evolution of Si-based fertilizers to impart growth and developmental effects of crop plants, such as enhancing photosynthesis and regulating electrolytic leakage under stress conditions (Chen et al., 2011). For example, Si enhances photosynthesis in mango trees and increases water and nutrient uptake under abiotic stress conditions (Santos et al., 2014). Si is found in almost all the plants in variable amounts, imparting varying physiological effects (Cooke and Leishman, 2011). Apart from its critical role in stress tolerance imposed by excess salt and drought, uptake of Si results in enhanced mechanical support to shoots and leaf blades (Chen et al., 2011; Zhu et al., 2019a). Numerous reports reveal the application of Si in mitigating abiotic and biotic stresses in various plant species (Zargar et al., 2019; Gou et al., 2020). Moreover, Si plays a vital role in ameliorating metal toxicity in several crop plants (Chen et al., 2011; Singh et al., 2019; Wu et al., 2019). Moreover, Si helps to mitigate metal ion stresses like that of aluminum (Al) and manganese (Mn) stress in crop plants (Tripathi et al., 2016). Water uptake through aquaporins and root hydraulic conductance is upregulated by administering exogenous Si (Liu P. et al., 2015). For instance, supplementation of Si in Talh trees (*Acacia gerrardii* Benth) induces salinity tolerance, possibly through overproduction of glycine betaine and proline to conserve water in tissues, positively regulating metabolic activity (Al-Huqail et al., 2019). Inclusively, Si is critical to plant growth and metabolism under a diverse range of abiotic stress conditions (Figure 1; Song et al., 2014; Yin et al., 2016).

Phytohormones play a pivotal role in stress tolerance by modulating a wide range of physiological, cellular, and molecular responses by activating various signal transduction pathways critical to defense mechanisms (Fahad et al., 2015a; Fei et al., 2019; Mubarik et al., 2021; Sabagh et al., 2021). These critical phytohormone-controlled physiological processes during defense include osmotic adjustment, plant-water relations, source/sink transition, the adjustment in photosynthesis, nutrient allocation, and activation of antioxidant defense systems (Miura and Tada, 2014; Khan et al., 2020a). The main phytohormones proven to mitigate the stress conditions through physiological, biochemical, and molecular adjustments include auxin, CK, ETH, GAs, SA, ABA, BRs, and JA (Khan et al., 2020a,b). The levels of phytohormones are reported to be enhanced upon exogenous Si supplementation under abiotic stress conditions (Kim et al., 2016). Considering the beneficial role of Si in alleviating the abiotic stress in crop plants, we believe it is the need of the hour to discuss its clinical properties at the physiological and molecular levels. Against this background, the current review is aimed to provide an in-depth account of the role of Si as a critical element in sustaining plant growth and productivity under abiotic stress conditions. We have also discussed the intervention of Si in triggering the phytohormone synthesis, regulating expression of genes in alleviating the abiotic stress conditions, and detailed the updated account on its role in enhancing growth and metabolism. We believe the current review will pave the way to further open the window of exploring this golden element and its frontline role in attaining global food security.

## Silicon – A Golden Key Element to Attain Sustainable Agriculture Growth

Population increase has put immense pressure on the agricultural system to overcome food insecurity. Even though diverse technological interventions increased the crop yield, at the same time, industrialization has triggered an increase in severity of environmental stresses, affecting crop production and, in turn, failure to achieve sustainable agriculture growth. Plants adjust to these factors by modulating their metabolic networks and inducing physiological changes (Raza et al., 2020a,b). Several reports suggest that Si plays an essential role in the production and yield of crops under extreme environmental conditions (Majeed Zargar et al., 2010; Liang et al., 2015b; Zargar et al., 2019; Salim et al., 2021). The growth and the metabolism of several monocots and dicot crops are enhanced by the active accumulation of Si in their organs in larger quantities (Liang et al., 2015c). High salinity results in reproductive disorders because higher Na^+^ competes with the transport of K^+^ and Ca^2+^ in the plasma membrane of cells (Zhu, 2016). Recent reports have suggested that Si alleviates Na^+^ accumulation, oxidative damage, and osmotic stress (Zhu et al., 2015; Flam-Shepherd et al., 2018; Yin et al., 2019). Several defense pathways related to phytohormone synthesis and compatible solutes are triggered by Si against abiotic stressors (Majeed Zargar et al., 2012; Mir et al., 2020). Elevated proline accumulation is observed in salt-sensitive genotypes of okra (*Abelmoschus esculentus*) upon exogenous application of Si to withstand salinity stress (Abbas et al., 2015). Si induced overproduction of total free amino acids, including proline and glycine betaine, in two *Capsicum annuum* cultivars, enhancing tolerance to salt stress (Pereira et al., 2013). Further accumulation of Si in wheat and soybean results in a declined trend of proline content, hence proving the efficacy of Si to induce its own mechanism to mitigate the salt stress (Lee et al., 2010; Bybordi, 2015).

**FIGURE 2:**
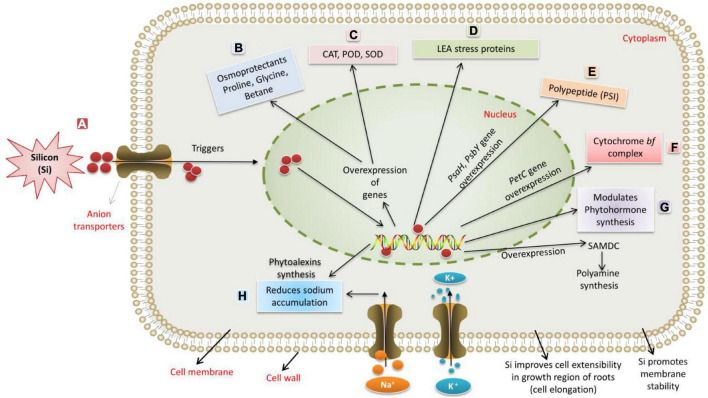
The silicon-induced mechanism for regulating abiotic stresses. **(A)** Silicon is taken up through anion transporters. **(B)** Drought and salinity stress induce osmotic stress and osmotic imbalance. Si enhances the synthesis of osmoprotectants like proline, glycine, and betaine. **(C)** Stress induces the formation of ROS. Si enhances the expression of CAT, SOD, and POD for protection against oxidative damage. **(D–F)** Si supplementation results in transcriptional regulation of genes related to photosynthesis, such as overexpression of *PsaH*, which encodes essential polypeptide subunits of photosystem-I (PSI) dimer, the *PsbY* (*Os08g02530*) gene encoding polyprotein component of Photosystem II and the *PetC* gene, encoding Rieske Fe-S center-binding polypeptide of cytochrome bf complex. **(G)** Si modulates the synthesis of plant growth regulators to alleviate stress. Si induces the S-adenosyl-L-methionine decarboxylase (SAMDC) gene, encoding essential enzymes responsible for synthesizing polyamines. **(H)** Salinity stress negatively affects plant growth by increasing the accumulation of ions up to toxic levels. Due to a higher influx of NaCl, plants experience more severe oxidative and ionic stress. Sodium-ion accumulation up to toxic levels triggers ROS production, which, in turn, severely damages the cellular components and accelerates senescence in mature leaves, leading to reduced growth and metabolism. Si administration improves uptake of K^+^, which, in turn, stimulates H+-ATPase enzymes in the plasma membrane that helps to overcome salt stress.

Si administration positively affects the growth, yield, and fruit quality of different fruit crops (Neumann and Zur Nieden, 2001). Si induces water and energy-saving capability, improvement in fruit quality, and an increase in yield. Due to the extent of differences in the Si accumulation, plants differ in their Si-based positive responses to abiotic stressors (Deren, 2001; Mitani and Ma, 2005; Keeping and Reynolds, 2009). Si is accumulated through specific transporters by major crop plants, including dicots like cotton, soybean, cucurbits, tomato, and monocots, such as rice, maize, and wheat, to alleviate different stress factors (Zargar and Agnihotri, 2013; Liang et al., 2015a). Application of Si leads to the overexpression of defense-related genes and their activity, apart from its role in accumulating defensive compounds, such as phytoalexins, momi-lactones, and phenolics (Liang et al., 2003; Rémus-Borel et al., 2005; Rahman et al., 2015). For instance, in rice plants, the ROS scavenging activities are decreased upon supplementation of Si during exposure to stressful conditions (Kim et al., 2014c). A clue to adapt Si as a remedial measure for sustainable agriculture is evident from the role of Si in the upregulation of genes involved in several adaptations, such as phytohormone metabolism and cell wall synthesis under stressful conditions in canola (*Brassica napus*) (Haddad et al., 2019). A wide range of genes has been modulated against the abiotic stress factors upon Si supplementation (Figure 2). Exogenous supply of Si alleviates sulfur and osmotic stress by promoting root growth *via* increased root sucrose levels in barley (Maillard et al., 2018). Emamverdian et al. (2018) reported that Si is capable of heavy metal remediation by initiating different pathways through scavenging ROS. It is reported that Si enrichment improved the growth and development of plants through enhancements in nutrient use efficiency (NUE) in winter wheat (Neu et al., 2017). For instance, Si is reported to decrease metal ion toxicity by reducing the solubility of metals in the cell wall or by detoxifying the metal ions (Wu et al., 2019; Vaculík et al., 2020). Several reports strongly report the role of Si to enhance tolerance to UV-B radiation by activation of defense mechanisms, fortification of the cell wall, etc., in crop plants (Yao et al., 2011; Guntzer et al., 2012). These reports suggest that Si plays a significant role in maintaining plants’ physiological and metabolic functions under drought stress conditions. In the preceding section, we have discussed the ameliorating effects of silicon on phytohormone-based resilience under extreme abiotic conditions.

## Si-Induced Differential Phytohormone Synthesis Under Abiotic Stress Conditions: An Update

Phytohormones play a critical role in growth and development and are essential to circumventing a diverse range of environmental stressors (Sato et al., 2010; Raza et al., 2019; Hafeez et al., 2021b; Mubarik et al., 2021). Phytohormones, such as auxin, CK, ABA, GAs, and BR, are vital to growth and metabolism during normal and stressful conditions (Sato et al., 2010; Raza et al., 2019; Hafeez et al., 2021b; Mubarik et al., 2021). These hormones modulate a wide range of physiological networks at low concentrations, once transported directly to the corresponding cells or transported to distant tissues (Colebrook et al., 2014), thus resilience to environmental stressors. The combinatorial effects of phytohormonal regulation, such as JA, ABA, and SA, play a vital role in mediating heat and drought tolerance in crop plants (Ilyas et al., 2021; Mostofa et al., 2021). Upregulation of GAs can ameliorate the abiotic stress conditions to maintain the growth and development of plants. In addition, upon exogenous application of SA, plants exhibit tolerance to various abiotic stresses like drought, heat, chilling, and tolerance to other stressors like viral, fungal, and bacterial attacks (Prakash et al., 2021; Saleem et al., 2021). Phytohormones like ABA are upregulated during osmotic stress to enhance tolerance by expressing several genes (Al Murad et al., 2020). Even though ABA levels were increased under salt stress, reverse trends were observed once Si was applied in soybean plants (Lee et al., 2010). ABA induces stomatal closure to prevent water loss under extreme saline conditions (de Ollas et al., 2013). In addition, ABA biosynthetic genes *ZEP* and *NCED3* are reported to be upregulated under abiotic stresses (Ma et al., 2018; Müller, 2021). On the other hand, ETH also senses external stimuli to adjust plant metabolism as per environmental conditions. It helps ameliorate abiotic stress in corroboration with other phytohormones, such as SA and JA (Rai et al., 2020). ETH regulates numerous physiological responses, such as triple response in the etiolated seedlings of a pea, induction of flowering, and seed germination (Kim et al., 2011). Higher levels of ETH are attributed to stress tolerance in plants, particularly tolerance to heat stress (Huang et al., 2021; Poór et al., 2021; Chen et al., 2022). Moreover, significant genes are upregulated by ETH to mediate diverse beneficial functions to plants (Kazan, 2015; Poór et al., 2021).

**FIGURE 3:**
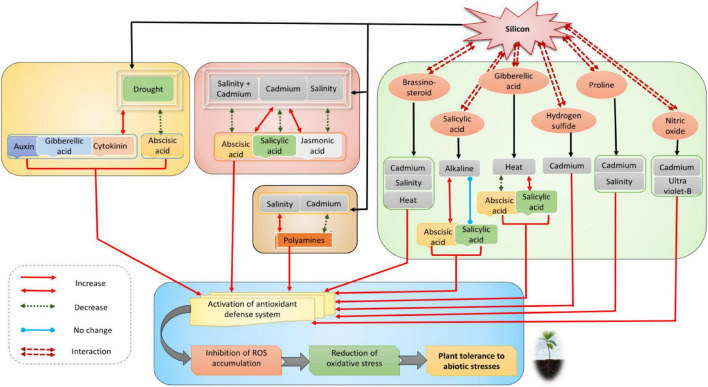
Interaction and crosstalk network of silicon with phytohormones and other signaling molecules in response to various abiotic and oxidative stresses. Read the text for further information.

On the other hand, Si interacts with several phytohormones and other signaling molecules, such as polyamines, hydrogen sulfide, and nitric oxide, in improving multiple abiotic stresses (Figure 3; Raza et al., 2021g; Sabagh et al., 2021; Tripathi et al., 2021). As a result, the interaction network improves the antioxidant defense system, reduces oxidative damage, and increases resistance to multiple abiotic stresses (Figure 3). In recent years, Si has been reported to enhance phytohormone synthesis to upregulate the physiological process of crop plants under extreme environmental conditions. Under abiotic stress conditions, Si enhanced the metabolism and synthesis of phytohormones in plants (Zhu and Gong, 2014; Kim et al., 2016). The major phytohormones upregulated by Si under abiotic stress conditions include ABA, JA, GA, ET, SA, BR, and IAA (Arif et al., 2021). Si-induced phytohormones synergistically regulate the growth, metabolism, and stress tolerance in pepper, as shown by *in silico* data analysis (Gómez-Merino et al., 2020). In tobacco and rice, Si regulates gene expression of plant hormones like ABA, JA, SA, and ETH to adjust metabolism against abiotic stress factors (Table 1). Lee et al. (2010), Kim et al. (2014c), Liang et al. (2015b) reported that Si-treated rice plants exposed to heavy metals increase ABA concentration, followed by a decrease after 14th day of treatment. In addition, reduction of SA’s and JA’s endogenous concentration has also been observed in these Si-treated rice plants. Various cellular and developmental processes, such as embryo development and dormancy, stomatal opening, and seed development, are regulated by ABA in plants (Sreenivasulu et al., 2012). Stress conditions trigger the upregulation of genes-encoding enzymes for the biosynthesis of ABA. In *Arabidopsis thaliana*, the signals from roots to regulate the decreased leaf water potential and conductance are accomplished by ABA triggered by the closure of stomata under drought stress (Hasan et al., 2021; Liu et al., 2021). ABA levels in Si-treated plants are decreased under salinity stress and increased when plants are exposed to heavy metals like cadmium (Cd) and copper (Cu) (Khan et al., 2021b; Mostofa et al., 2021; Tripathi et al., 2021).

**TABLE 1:** Silicon-induced gene expression in plants under various abiotic stress conditions.

Abiotic stress	Plant name	Genes	Gene function/Protein encoded	References
Drought stress	*Arabidopsis thaliana*	*At5g22460*	Esterase lipase thioesterase family protein Transporter gene	Li et al., 2008; Naz et al., 2021a
	*Arabidopsis thaliana*	*At5g59030*	Copper transporter gene	Li et al., 2008; Carneiro et al., 2017
	*Oryza sativa*	*AK070732*	Member of RING domain-containing protein family Regulatory gene	Khattab et al., 2014; Malik et al., 2021
	*Oryza sativa*	*AF300971*	Dehydration responsive element binding protein Regulatory gene	Khattab et al., 2014; Malik et al., 2021
	*Oryza sativa*	*AJ578494*	Choline monooxygenase Regulatory gene	Khattab et al., 2014; Malik et al., 2021
	*Oryza sativa*	*AB028184*	NAC regulons (No apical meristem (NAM), Arabidopsis thaliana activating factor [ATAF], and cup-shaped cotyledon [CUC]) Regulatory gene	Khattab et al., 2014; Manivannan and Ahn, 2017
	*Oryza sativa*	*NM-001074375*	Dehydrin Regulatory gene	Khattab et al., 2014
	*Sorghum bicolor*	*Sb02g025110*	S-Adenosyl-L-methionine decarboxylase Polyamine synthesis	Yin et al., 2016; Malik et al., 2021; Wang et al., 2021
	*Sorghum bicolor*	*Sb04g025720*	S-Adenosyl-Metdecarboxylase Polyamine synthesis	Yin et al., 2016
	*Sorghum bicolor*	*Sb06g021540*	S-Adenosyl-Metdecarboxylase Polyamine synthesis	Yin et al., 2016
	*Sorghum bicolor*	*Sb10g002070*	Arginine decarboxylase Polyamine synthesis	Yin et al., 2016
	*Sorghum bicolor*	*SbPIP1;6*	Plasma membrane intrinsic protein (PIP) 1;6 aquaporins	Liu P. et al., 2015
	*Sorghum bicolor*	*SbPIP2;6*	plasma membrane intrinsic protein (PIP) 2;6 aquaporins	Liu P. et al., 2015
	*Sorghum bicolor*	*SbPIP2;2*	plasma membrane intrinsic protein (PIP) 2;2 aquaporins	Liu P. et al., 2015
	*Sorghum bicolor*	*Sb04g021790*	N-Carbamoyl putrescine amidohydrolase Polyamine synthesis	Yin et al., 2016
	*Oryza sativa*	*OsRDCP1*	Oryza sativa RING domain-containing protein	Khattab et al., 2014
	*Oryza sativa*	*OsCMO*	Oryza sativa choline monooxygenase	Khattab et al., 2014; Rehman et al., 2021
	*Oryza sativa*	*LEA3*	Late Embryogenesis Abundance protein	Khattab et al., 2014
	*Oryza sativa*	*DREB2A*	Dehydration-responsive element-binding protein 2A	Khattab et al., 2014
	*Oryza sativa*	*OsRAB16b dehydrin*	Dehydrin RAB16B protein	Khattab et al., 2014
Cold stress	*Oryza sativa*	*SAMDC*	S-adenosyl-L-methionine decarboxylase	Pottosin and Shabala, 2014
	*Arabidopsis*	*DREB1A*	Dehydration-responsive element-binding protein 1A	Liu et al., 1998; Liang et al., 2021
	*Arabidopsis*	*DREB1B*	Dehydration-responsive element-binding protein 1B	Liu et al., 1998; Manivannan and Ahn, 2017
	*Arabidopsis*	*DREB1C*	Dehydration-responsive element binding protein 1C	Liu et al., 1998; Manivannan and Ahn, 2017
Salinity stress	*Lycopersicum esculentum*	*leDREB-1*	Lycopersicum esculentum Dehydration-responsive element binding-1 protein	Muneer and Jeong, 2015; Dhiman et al., 2021
	*Lycopersicum esculentum*	*leDREB-2*	Lycopersicum esculentum Dehydration-responsive element binding-2 protein	Muneer and Jeong, 2015
	*Lycopersicum esculentum*	*leDREB-3*	Lycopersicum esculentum Dehydration-responsive element binding-3 protein	Muneer and Jeong, 2015; Dhiman et al., 2021
	*Oryza sativa*	*ZEP*	zeaxanthin epoxidase	Kim et al., 2014c
	*Oryza sativa*	*NCED1*	9-cis-epoxicarotenoid oxygenase 1	Kim et al., 2014c
	*Oryza sativa*	*NCED4*	9-cis-epoxicarotenoid oxygenase 2	Kim et al., 2014c
	*Lycopersicum esculentum*	*leLsi-1*	Lycopersicum esculentum Low Si-1 transporter	Muneer and Jeong, 2015
	*Lycopersicum esculentum*	*leLsi-2*	Lycopersicum esculentum Low Si-2 transporter	Muneer and Jeong, 2015
	*Lycopersicum esculentum*	*leLsi-3*	Lycopersicum esculentum Low Si-3 transporter	Muneer and Jeong, 2015
	*Lycopersicum esculentum*	*leAPX*	Lycopersicum esculentum Ascorbate peroxidase	Muneer and Jeong, 2015
	*Lycopersicum esculentum*	*leSOD*	Lycopersicum esculentum Superoxide dismutase	Muneer and Jeong, 2015
	*Lycopersicum esculentum*	*leCAT*	Lycopersicum esculentum Catalase	Muneer and Jeong, 2015
	*Glycine max*	*TaLsi1*	Si-Influxer	Hwang et al., 2007
	*Glycine max*	*TaLsi2*	Si-effluxer	Hwang et al., 2007
	*Oryza Sativa*	*AOC*	Allene oxide cyclase	Kim et al., 2014c
	*Oryza Sativa*	*AOS*	13-Allene oxide synthase (13-AOS)	Kim et al., 2014c
	*Oryza Sativa*	*OPR3*	Opda reductase3	Kim et al., 2014c
	*Oryza Sativa*	*LOX*	Lipoxygenase	Kim et al., 2014c
Metal ion stress	*Oryza Sativa*	*OsHMA3*	Oryza Sativa heavy metal ATPases	Kim et al., 2014a
	*Oryza Sativa*	*OsLsi-1*	Oryza Sativa Low Si Rice 1	Kim et al., 2014a
	*Oryza Sativa*	*OsLsi1*	Oryza Sativa Low Si Rice 2	Kim et al., 2014a
	*Oryza Sativa*	*OsHMA2*	Oryza Sativa heavy metal ATPases	Kim et al., 2014a
	*Oryza sativa*	*Os08g02630*	Subunit of oxygen evolving complex-PSII Photosynthesis	Song et al., 2014; Lesharadevi et al., 2021
	*Oryza sativa*	*Os05g48630*	Photosynthetic co8y stability maintenance Photosynthesis	Song et al., 2014; Lesharadevi et al., 2021
	*Oryza sativa*	*Os07g37030*	Maintenance of cytochrome Photosynthesis	Song et al., 2014
	*Oryza sativa*	*Os03g57120*	Ferrodoxin NADP + reductase Photosynthesis	Song et al., 2014; Lesharadevi et al., 2021
	*Oryza sativa*	*Os09g26810*	Subunit of LHC II complex Photosynthesis	Song et al., 2014; Lesharadevi et al., 2021
	*Oryza sativa*	*Os04g38410*	Subunit of LHC II complex Photosynthesis	Song et al., 2014; Lesharadevi et al., 2021
	*Oryza Sativa*	*PetC gene*	Rieske Fe-S center-binding polypeptide of cytochrome bf complex	Song et al., 2014; Lesharadevi et al., 2021
Heat stress	*Solanum lycopersicum* L.	*SlLsi1*	Low Si Rice 2	Khan et al., 2020b
	*Solanum lycopersicum* L.	*SlLsi2*	Low Si Rice 2	Khan et al., 2020b
	*Solanum lycopersicum* L.	*SlHsfA1a-b*	Heat Stress Transcription Factor	Khan et al., 2020b
	*Solanum lycopersicum* L.	*SlHsfA2-A3*	Heat Stress Transcription Factor	Khan et al., 2020b
	*Solanum lycopersicum* L.	*SlHsfA7*	Heat Stress Transcription Factor	Khan et al., 2020b
	*Solanum lycopersicum* L.	*SlDREB2*	dehydration-responsive element binding proteins	Khan et al., 2020b
Wound stress	*Lycopersicum esculentum*	*OsAOS1*	Allene oxide synthase-1	Kramell et al., 1995; Agrawal et al., 2003
	*Lycopersicum esculentum*	*OsAOS2*	Allene oxide synthase-1	Kramell et al., 1995; Agrawal et al., 2003
	*Lycopersicum esculentum*	*OsOPR3*	12-oxo-phytodienoic acid reductase	Kramell et al., 1995; Agrawal et al., 2003
	*Lycopersicum esculentum*	*OsLOX*	Lipoxygenase	Kramell et al., 1995; Agrawal et al., 2003
Chilling and drought	*Oryza sativa*	*OsDREB1*	*Oryza sativa* Dehydration-responsive element-binding protein 1A	Ito et al., 2006
Salinity and drought stress	*Arabidopsis*	*DREB2B*	Dehydration-responsive element-binding protein 2A	Islam and Wang, 2009
Sulfur deficiency and osmotic stress	*Hordeum vulgare*	*ZEP*	Zeaxanthin epoxidase	Maillard et al., 2018
Sulfur deficiency and osmotic stress	*Hordeum vulgare*	*NCED3*	9-cis-epoxicarotenoid oxygenase 1	Maillard et al., 2018
Salinity/Drought stress	*Oryza sativa*	*NAC5*	NAC (NAM/ATAF/CUC) transcription factor	Khattab et al., 2014

Moreover, Si-treated plants ameliorate metal toxicity, leading to low production of ABA. Time-dependent effects of Si on ABA and JA synthesis were also reported under abiotic stressors (Kim et al., 2014c). Overexpression of Si transporter genes, such as *TaLsi1* (Si-Influxer) and *TaLsi2* (Si-effluxer), under salinity-induced ABA was reported upon supplementation of exogenous Si in wheat (Zia et al., 2021). Si-induced alteration of ABA-related genes glyceraldehydes-3-phosphate dehydrogenase (GAPDH), cyclophilin (CYC), ADP-ribosylation factor (ADP-RF1), and sulfur transporter (HsST1) alleviates sulfur deficiency and osmotic stress in crop plants (Maillard et al., 2018). Moreover, ABA levels are declined due to downregulation of genes by exogenous supplementation of Si to enhance the stress tolerance in barley (Maillard et al., 2018). It is reported that, in rice plants exposed to salt stress, Si upregulated expression of ABA biosynthesis genes, such as *ZEP*-encoding zeaxanthin epoxidase, *NCED1*-encoding 9-cis-epoxicarotenoid oxygenase 1, and *NCED4*-encoding 9-cis-epoxicarotenoid oxygenase 2 (Kim et al., 2014b). A versatile transcription factor, NAC encoded by *RD25* genes, was found to be induced by stress factors, such as salinity and drought, in addition to its induction upon treatment of crop plants by JA and ABA (Singh et al., 2021).

JA is another hormone critical to generate a response against a wide range of stress factors through activation of defense signaling pathways (Pieterse et al., 2012; Caarls et al., 2015). JA production is lowered upon exogenous treatment of Si; later, decline in JA can be correlated by low-stress levels exerted by Si treatment (Wang et al., 2020; Raza et al., 2021b). The reduction in the levels of JA induced by Si involves the modulation of several signaling pathways (Liu and Timko, 2021; Tripathi et al., 2021). Si application drastically improves the signaling of JA to initiate a defense system during wounding stress in rice plants (Hamayun et al., 2010). The expression of genes related to JA synthesis, like *LOX* and other genes, is upregulated when Si is administered to wound-stressed rice plants (Kim et al., 2011, 2014b). Under wound stress *via* feedforward activation, the *LOX* gene further amplifies the expression of JA in *A. thaliana* (Sato et al., 2010). Genes related to JA like *OsAOS1*, *OsAOS2*, *OsOPR3*, and *OsLOX* were downregulated upon exogenous application of Si during wound stress, highlighting the role of Si in triggering metabolic networks to heal the mechanical injuries in rice plants (Kim et al., 2014b). In addition, overexpression genes like *AOC, AOS, OPR3*, and *LOX* during early stages occurred during wound stress; downregulation of JA biosynthesis was also demonstrated in tomatoes. GAs are an important class of phytohormones critical to plants, such as seed germination and fruiting (Colebrook et al., 2014). The endogenous levels of GA are upregulated under salinity stress in *Glycine max* upon application of Si at 2.5-mM concentration (Arif et al., 2021; Dhiman et al., 2021). The Si-triggered upregulation of GA_1_ and GA_4_ content in cucumber plants helps to withstand drought and salinity stress (Hamayun et al., 2010). The combinatorial effect of Si and nitrogen on the growth and metabolism of two rice cultivars led to upregulation of GA_20_ and GA_1_, initiating vegetative and anthesis stages (Lee et al., 2010). Moreover, the decreased levels of GA under salt stress were restored by applying Si in rice plants (Lee et al., 2010). In another study, Si-mediated increase in the synthesis of GA, such as GA_1_ and GA_4_ levels, profoundly enhanced salt tolerance in *Glycyrrhiza uralensis* Fisch (Zhang et al., 2018). Likewise, Si-treated plants enhanced GA concentration in saline conditions and reduced JA concentration under wound response (Lee et al., 2010; Kim et al., 2011).

Auxin is another phytohormone critical to a wide range of development and growth of plants (Du and Scheres, 2018). It has been reported in several studies that IAA plays an essential role in the tolerance of crop plants to salinity stress (Fahad et al., 2015b; Hussain et al., 2019). Activation and upregulation of numerous genes triggered by auxins have been reported in rice, soybean, and Arabidopsis, which are important for the development and growth of plants (Wani et al., 2016). It is reported that, upon administration of Si, IAA levels decreased in crop plants, resulting in a delay of leaf senescence in canola (*Brassica napus*) (Haddad et al., 2018). The synthesis of IAA and ABA was transcriptionally downregulated by Si supplementation, resulting in osmotic stress tolerance in barley (Maillard et al., 2018).

Likewise, Si applied to the rice plants having wound stress resulting in decreased ETH production compared to rice plants not treated with Si (Kim et al., 2011). The delaying of senescence was observed in crop plants upon Si supplementation through increased synthesis of CK biosynthesis in Arabidopsis and sorghum (Markovich et al., 2017). Si enhanced the growth and development of rice plants by its effect on endogenous ETH and JA levels, even though JA and JA show inverse correlation under wound stress (Kim et al., 2011). SA, another potential phytohormone, has important physiological implications on crop plants, such as transport and uptake of ions, photosynthetic metabolism, and membrane permeability during stress (Noreen et al., 2009). More importantly, SA is reported to outwit abiotic stressors like the generation of ROS, attacks by pathogens, salinity, and drought stress (Hara et al., 2012). Tolerance to stress conditions, such as salinity, is initiated by SA-based interplay of phytohormones (El-Mashad and Mohamed, 2012). Combinatorial supplementation of SA and Si reduces damage caused by boron toxicity through increased carotenoid, chlorophyll content, biomass production, and reduction in oxidative stress (Eraslan et al., 2008). A possible mechanism of stress tolerance induced by SA occurs through expression of several genes, such as those coding for pathogenesis-related proteins (PR-proteins) (Kidokoro et al., 2009). Exogenous application of SA helps in the resistance and adaptation of crop plants in various abiotic stress factors, such as drought, salinity, and iron deficiency (Mostofa et al., 2021; Tariq et al., 2022). In pea plants, abiotic stress, such as oxidative stress, was reported to be remedied by exogenous supplementation of Si and SA (Rahman et al., 2017). Moreover, soybean plants withstand salt stress through modulation of SA and JA upon treatment with Si (Kim et al., 2014a). In conclusion, the reports suggest that interplay of Si and phytohormones is critical to the growth and metabolism of plants under drought stress conditions.

## Physiological Benefits of Silicon Under Abiotic Stress

The growth and the metabolism of plants are improved through their mechanisms to develop tolerance against abiotic stressors. Later attributes were primarily alleviated by supplementation of Si to withstand negative environmental stress factors (Arif et al., 2021; Souri et al., 2021). In sugarcane, Si has been attributed to enhancing leaves’ rigidity and erectness, resulting in improved photosynthesis (Verma et al., 2019). Pfannschmidt and Yang (2012) reported overexpression of *PsaH*, which encodes essential polypeptide subunits of photosystem-I (PSI) dimer upon supplementation of Si. A comparative study showed that Si-administered cucumber exhibited increased perception of light, the enhanced concentration of chlorophyll pigments, and rough petioles (Adatia and Besford, 1986; Lozano-González et al., 2021; Meng et al., 2021). Several reports suggest that Si positively modulates the plant growth and yield in rice plants (Dorairaj et al., 2017; Garg and Singh, 2018). It has been demonstrated that Si positively modulates the growth, development, and metabolism in sugarcane (*Saccharum officinarum*) (Verma et al., 2021), beans (*Phaseolus vulgaris*) (Zuccarini, 2008; Nosrati et al., 2014; El-Saadony et al., 2021), cotton, (*Gossypium hirsutum*) (Wang et al., 2001; dos Santos et al., 2020), rice (Hwang et al., 2007; Feng et al., 2011), and soybean (*Glycine max)* (Hamayun et al., 2010).

Moreover, Si has been shown to positively modulate the metabolism of several crop plants, such as spinach (*Spinacia oleracea*) (Eraslan et al., 2008), wheat (Gong et al., 2005; Sattar et al., 2019; Bukhari et al., 2021a), barley (Savant et al., 1999; Benslima et al., 2021; Sierra et al., 2021), and maize (Liang et al., 2005; El-Mageed et al., 2021). Shi et al. (2013) reported that tomato seedlings completely recover their growth and photosynthesis once exposed to Si under water stress conditions. Si can ameliorate the metal ion toxicity and, in turn, able to enhance the quality and quantity of biomass in *Phaseolus vulgaris* (Zuccarini, 2008; Artyszak, 2018). The application of Si detoxifies Na^+^ through modification of the antioxidant defense system. Later effect positively modulates important biochemical and physiological metabolic networks in sunflower and sorghum under abiotic stresses, particularly salt stress, to increase shoot dry matter production (Hurtado et al., 2020). Si-treated rice plants significantly improved the shoot, and root morphology restored photosynthetic efficiency amid Cd toxicity (Bari et al., 2020). In addition, Si was reported to mediate a wide range of physiological benefits like enhanced growth, nutrient uptake, provision of mechanical strength, and resistance to biotic stressors like fungal diseases (Akhtar and Hussain, 2008; Bakhat et al., 2018; Souri et al., 2021). Moreover, Si helps to reduce lipid peroxidation in crop plants exposed to wound stress in rice plants (Kim et al., 2014b). The germination characteristics like the germination rate and percentage and shoot length are highly enhanced by applying Si under normal and drought and salinity stress conditions in tomatoes (Shi et al., 2014). In crop plants, such as maize, rice, wheat, tomato, and cucumber, Si enhanced shoot and root growth under salt stress (Wu et al., 2015). Water balance in cucumber is improved by exogenous supplementation of Si through its effect on increasing the root-shoot ratio under salt stress (Wang et al., 2015). For instance, the supplementation of Si leads to the overexpression of stress-responsive genes, such as *Oryza sativa* RING domain-containing protein *(OsRDCP1*), *Oryza sativa* choline monooxygenase (*OsCMO*), *DREB2A*, dehydrin *OsRAB16b*, and *NAC5*, under abiotic stress conditions (Khattab et al., 2014). Production of ROS is disastrous to crop plants as it induces lipid peroxidation (Gill and Tuteja, 2010). The foliar application of Si in okra leads to overexpression of ROS-scavenging genes coding for CAT, POD, and SOD during salinity stress. Later, enzymes help decrease the lipid peroxidation in okra plants (Abbas et al., 2015). Differentially enhanced activity of ROS-scavenging enzymes, such as APX, CAT, and POD in roots, shoots, and leaves, was reported in alfalfa under salinity stress by application of Si (Wang et al., 2010).

Moreover, Si reduces oxidative stress in tomato plants under salinity stress, indicating the effect of Si on the activity of antioxidant enzymes (Li et al., 2015). Similar results were also observed in rice plants grown under salinity stress when Si was applied (Kim et al., 2014c). Numerous studies back up the role of Si in alleviating abiotic stress in crop plants (Mir et al., 2019). The combined ameliorative effect of Si ad *Bacillus subtilis* fbl-10 was found to enhance tolerance to Pb (Lead) toxicity in brinjal (*Solanum melongena*) (Shah et al., 2021). Moreover, the effect of Si on delaying leaf senescence in mustard (*Brassica juncea*) under drought and salinity stress was accomplished by modulation of homeostasis of ions and antioxidative defensive systems (Alamri et al., 2020). It is reported that, after deposition, Si helps to thicken the xylem cell wall and the Casparian strips of endodermis to impede the metal ion transport in crop plants (da Cunha and do Nascimento, 2009; Mandlik et al., 2020; Souri et al., 2021). In combination with SA, in mung bean seeds, Si enhanced physiological parameters through the decreased accumulation of Na^+^ and increased proliferation of K^+^ (Lotfi and Ghassemi-Golezani, 2015). Several physiological parameters, such as the transpiration rate, stomatal conductance, chlorophyll levels, and membrane permeability, are maintained by Si during abiotic stress, particularly during salinity stress (Coskun et al., 2016). Besides, the adverse effects of heat stress in tomato plants are mitigated by exogenous application of Si, further enhancing biomass production and shoot length (Khan et al., 2020b). Plants overexpress several genes pertaining to transpiration, photosynthesis, synthesis of polyamines, and secondary metabolism in response to abiotic stressors (Abdel-Haliem et al., 2017). For instance, in sorghum, it is reported that, upon application of Si, the root growth was stimulated by root elongation due to cell wall extensibility in the growth region (Hattori et al., 2003; de Oliveira et al., 2019). Several reports suggest that Si induces modulation of phytohormones to delay senescence in rice and sorghum (Lee et al., 2010; Kim et al., 2014c; Yin et al., 2014). In chickpea cultivars, drought tolerance was regulated by supplementation of Si through improvement of various growth parameters. High salinity in rice and Mn, Al, Cu, and Cd toxicity in sorghum and wheat is alleviated by supplementation of Si (Kang et al., 2015; Keller et al., 2015; Rizwan et al., 2015).

A large number of stress-tolerant genes are upregulated by exposure of crop plants to Si. For instance, studies have reported Si results in transcriptional regulation of genes related to photosynthesis during zinc stress in rice plants (Song et al., 2014). In rice, wide ranges of stressful conditions are acclimatized by the expression of LEA genes-encoding stress tolerance LEA proteins (Lenka et al., 2011). Several studies report overexpression of the *OsNAC5* gene is attributed to alleviating the stress tolerance through the abundant expression of LEA3 in rice plants (Takasaki et al., 2010). Likewise, in rice, upregulation of *OsRDCP1* and *OsCMO* gene levels alleviated the dehydration stress upon supplementation of exogenous Si (Khattab et al., 2014). The overexpression of the S-adenosyl-L-methionine decarboxylase (*SAMDC*) gene, encoding vital enzymes responsible for the synthesis of polyamines, is augmented by supplementation of Si (Pottosin and Shabala, 2014). These polyamines are critical to withstand the onslaught by environmental stressors (Liu P. et al., 2015). The wonderful effects of Si under drought stress conditions have considerably shifted scientists’ focus on its emerging role in maintaining the physiological hemostasis of crop plants. In the preceding sections, we have discussed the role of Si in alleviating the stress tolerance of plants against salinity, drought, heavy metal, temperature, and other abiotic stressors.

## Salinity Tolerance Mediated by Silicon

Agricultural crop production is grossly limited by salinity stress leading to a drastic decline in food production at the commercial and industrial levels (Munns and Gilliham, 2015). Plants are negatively affected by salinity through an increase in ionic and osmotic stress. On a global scale, almost 6% of cultivated land is graded as saline and is expected to increase by 2050 due to global climatic change, further worsening the scenario of crop production. Moreover, salinity stress induces a reduction in osmosis, imbalance in nutrient supply, and enhanced accumulation of ions up to toxic levels (Hasegawa et al., 2000; Hafeez et al., 2021a; Saddiq et al., 2021). Further, it is evident that salt stress is a major abiotic factor that inhibits crop plants’ growth and metabolism, resulting in reduced crop yield (Han et al., 2021). The elevation of salt stress is further compounded by anthropogenic activities, such as saline water irrigation, pollution through industries, and higher use of chemical fertilizers (Zhu et al., 2019a). Sodium-ion accumulation up to toxic levels triggers the production of ROS, which, in turn, severely damages the cellular organelles, such as chloroplast, cell membrane, mitochondria, and peroxisomes, to impair the metabolic networks in crop plants (Munns and Gilliham, 2015).

Moreover, it is reported that the accumulation of Na^+^ to toxic levels in older leaves accelerates senescence in mature leaves, leading to reduced growth and metabolism (Munns and Gilliham, 2015; Saddiq et al., 2021). Global production of agricultural food crops is reduced up to 20% by salinity stress among all the abiotic stresses (Kumar et al., 2017; Hussain et al., 2018). High salinity reduces the potential of plants to absorb the water, resulting in a reduction of cell expansion and low stomatal activity due to less inter- and intracellular water levels. Due to a higher influx of NaCl, plants experience more severe oxidative and ionic stress (Ferchichi et al., 2018). The salt stress is attenuated more frequently by Si administration (Etesami and Jeong, 2018; Liu et al., 2019). In maize, salinity stress is alleviated by exposure to Si through improvements in growth and maintaining ion homeostasis *via* decline in Na^+^ uptake (Ali et al., 2021). Si improves uptake of K^+^, which, in turn, stimulates H^+^-ATPase enzymes in the plasma membrane to overcome salt stress in maize and other plants (Liang et al., 2005; Liu et al., 2019; Dhiman et al., 2021). Under salinity stress, the application of Si in rice was reported to decrease the accumulation of sodium and chloride ions through apoplastic transport (Shi et al., 2013). Hyper-accumulators of Si were found to form double cuticle layers, enhancing photosynthesis by preventing evapotranspiration. Moreover, Si influences starch metabolism, glycolytic pathways, and the Krebs cycle under osmotic stress in barley (Hosseini et al., 2017). In addition, cucumber seedlings could ameliorate the extreme oxidative stress upon the influx of Si (Gou et al., 2020). Declined chlorophyll content due to salinity stress was reported to be significantly ameliorated by the supplementation of exogenous Si to root zones or foliar in tomato (Al-aghabary et al., 2005), pea (Ahmad and Jhon, 2005), and cucumber (Hamayun et al., 2010) crop plants. In plants, Si accumulates and deposits in discrete silica bodies in the form of phytoliths, usually beneath the cell walls on different parts, such as roots, stems, and leaves. These phytoliths then bind with Na^+^ to reduce their uptake and increase K^+^ uptake to different regions of plants. Concomitantly, it is reported that Si induces greater H + -ATPase activity to lower the electrolyte leakage resulting in higher secretion of Na^+^ from cells in *Puccinellia distans* (Soleimannejad et al., 2019). Furthermore, to avoid multiple abiotic stressors, Si deposition in leaf blades during salinity results in converting complexions to non-toxic stable ions to regulate ROS production (Mandlik et al., 2020; Ahire et al., 2021; Dhiman et al., 2021). Due to the deposition of Si in the cell walls of endodermis and rhizodermis, the transport of Na^+^ is blocked through a reduction in apoplastic movement of Si across the root system, leading to a drastic decline in the transport of Si from roots to shoots, hence salinity tolerance (Khan et al., 2019). Moreover, negative impacts of higher salt levels are greatly tolerated by Si application through the production of glycine betaine to maintain metabolic activity to regulate water levels in plants (Al-Huqail et al., 2019). The compatible osmolytes, such as glycine betaine, are critical to the stability of membrane, functional proteins, protein complexes, and enzymes under saline conditions (Rajasheker et al., 2019). In addition, Si enhances proline content to induce salt tolerance in cucumber (Gou et al., 2020). Plants synthesize proline as major osmoprotectants that protect the integrity of membrane and protein stability under salinity stress (Ahanger et al., 2020a). Moreover, the variable concentration of Si resulted in enhanced activities of enzymes, such as glutamine synthetase (GS), nitrate reductase (NR), sucrose synthetase (SS), and sucrose phosphate synthetase (SPS), to enhance nitrogen and carbon metabolism under salinity stress in *Glycyrrhiza uralensis* Fisch (Cui et al., 2021). In tomato, several genes, such as *leDREB-1*, *leDREB-2*, *leDREB*-3, *leLsi*-1, *leLsi*-2, *leLsi*-3, *leAPX*, *leSOD*, and *leCAT*, are upregulated upon supplementation of Si upon exposure to salt stress (Ngwepe et al., 2019), whereas the exposure of *Arabidopsis* to salinity and dehydration resulted in transient expression of *DREB2A* and *DREB2B* (Liu et al., 1998; Sakuma et al., 2006; Manna et al., 2021; Singh and Chandra, 2021). The increase in contents of minerals, such as Ca and Mg in tomato leaves and Mg, K, Ca, and P in *Trifolium alexandrinum*, was reported upon supplementation of Si to regulate growth under salt stress (Abdalla, 2011; Li et al., 2015). Overall, Si plays a critical role in circumventing salt stress through the induction of diverse biochemical and physiological mechanisms (Al Murad et al., 2020). See Table 2 for some recent examples of Si-induced salinity tolerance.

**TABLE 2:** A summary of some recent reports on silicon-induced abiotic stress tolerance in crop plants.

Plant specie	Stress condition	Silicon dose	Protective role	References
**Salinity**				
*Zea mays* L.	80 mM NaCl; 160 mM NaCl	Si (Na_2_SiO_3_; 1 mM)	Si addition alleviated the salt toxicity by increasing RWC, MSI, CAT, SOD, APX, and POD activities	Ali et al., 2021
*Oryza sativa* L.	200 mM NaCl	2 mM K_2_SiO_3_	Si improves the rice growth and salinity tolerance by modulating the Salt-Overly Sensitive (SOS) pathway	Gupta et al., 2021
*Medicago sativa* L.	120 mM NaCl	Si 3 mM	Si application lowered the oxidative damage, modulated SOD, polyphenol oxidase activities, and improved flavonoid, total polyphenol, and carotenoid contents	El Moukhtari et al., 2021
*Solanum tuberosum* L.	NaCl 5, 8, and 12 dS m^–1^	NaSiO_3_- NPs 400 mg L^–1^, SiO_2_ 1000 mg L^–1^	Si application increases the proline content and leaf soluble carbohydrates than control Si treatment also lowered the Na^+^/K^+^ twice and induced antioxidant enzyme activities	Kafi et al., 2021
*Oryza sativa* L.	0, 25, 50, and 100 mM NaCl	Si, 2 mM	Si application improves the endogenous levels of polyamine by upregulating PAs biosynthetic enzymes While reducing GABA accumulation by downregulating PAs catabolism and maintaining functional GABA shunt, which may lower the oxidative damage	Das et al., 2021
*Cucumis sativus* L.	75 mM NaCl	1.5 mM Si	Si treatment increases the numerous growth-associated parameters and alleviates the adverse salinity effects. Si application reduces the chlorides (Cl^–^) shoot concentration	Kaloterakis et al., 2021
**Drought**				
*Arachis hypogaea* L.	Solitary drought (i.e., 10% PEG and 15% PEG)	Na_2_SiO_3_ 2 mM	Si treatment promotes mineral nutrient absorption improves RWC, leaf chlorophyll content, and biomass It also provides osmoprotection by accumulating metabolites and improving the JA, IAA, GA3, zeatin levels	Patel et al., 2021
*Avena sativa* L.	June, only 6 mm rainfall despite a 30-year average of 66.5 mm	Si 3.0 L ha^–1^	Si treatment increased the synthesis rate (16.8–149.3%), transpiration (5.4–5.6%), air–leaf temperature difference (16.2–43.2%), Chl (1.0%) and carotenoid (2.5%) content	Kutasy et al., 2022
*Zea mays* L.	10% (w/v) PEG	1.0 mM Na_2_SiO_3_	Si treatment decreased the electrolyte leakage from 0.64 to 0.52% and increased membrane stability 12%, Chl a 35%, Chl b 31%, and carotenoids 51% than control	Bijanzadeh et al., 2022
*Zea mays* L.	Full irrigation 100% and deficit irrigation 80%	Na_2_SiO_3_ 0, 2, and 4 mM at 40 and 60 days after planting	Si treatment improves plant growth, gaseous exchange, cell membrane integrity, water use efficiency, physiological performance, and maize productivity It also lowered the concentrations of Ni^+2^, Cd^+2^, and Cr^+3^ in leaves and grains of maize	El-Mageed et al., 2021
*Triticum aestivum* L.	Samples drying in an oven at 70°C for 72 h	Si, 6 mM	Si treatment alleviated the oxidative stress and negative drought impacts by increasing the antioxidant enzyme activities	Naz et al., 2021a
*Cucumis melo* L.	soil moisture regimes 100, 75, and 50% FC	H_4_SiO_4_, 0, 100, 200, and 400 kg ha^–1^	Si treatments increased self-resistance to lodging and strengthened cell wall, restricted fungal disease, and insect infestations, reduced mutual shading, improved water balance, reduced transpiration, and water loss	Alam et al., 2021
**Toxic metals/metalloids**				
*Triticum aestivum* L.	Cd 25 mg kg^–1^	3 mM Si	Si treatment reduced the Cd-mediated oxidative stress and improved photosynthetic pigments, net photosynthetic rate, strengthening the antioxidant defense system, enhancing metabolite accumulation, and improving plant nutrient status	Thind et al., 2020
*Triticum aestivum* L.	As(V) 25, 50, and 100 μM	5 mM Si	Si treatment mitigated the arsenate-induced effects and oxidative stress Si application also respiratory cycle, GABA synthesis, and its associated enzymes	Sil et al., 2018
*Capsicum annuum* L.	0.05 mM B, 2.0 mM BT	2.0 mM Si	Si treatment improves plant growth, proline content, and various antioxidant enzymes activities while lowering the MDA, H_2_O_2_ contents, and membrane leakage	Kaya et al., 2020
*Poa annua* L.	100 μM Cd	1 mM Si	Si application alleviated the Cd-toxicity, restored the activity of G6PDH and the expression of G6PDH, and lowered the oxidative stress induced by Cd	Li et al., 2017
*Brassica napus* L	0.5 and 1.0 mM CdCl_2_	SiO_2_, 1.0 mM	Si treatment reduced the H_2_O_2_ and MDA contents and improved antioxidant defense mechanisms through increasing the AsA and GSH pools and activities of AsA-GSH cycle and glyoxalase system enzymes and CAT	Hasanuzzaman et al., 2017
*Zea mays* L.	Ni 100 μM	2.5 mM Si	Si treatment mitigated the Ni-induced stress by enhancing membrane stability and influencing enzymatic (SOD, POX, and CAT) and non-enzymatic (Pro, and AsA) defense systems	Fiala et al., 2021
*Salvia officinalis* L.	400 μM Cu	0, 0.25, 0.5, and 1 mM Si	Si treatment alleviated the oxidative damage, increasing proline content, enhancing the CAT and SOD activities, and up-regulating the *SOD* gene expression	Pirooz et al., 2021
**Temperature (cold/heat)**				
*Solanum lycopersicum* L.	30°C to 43 ± 0.5°C	1 Mm Si	Si application provides thermotolerance by activating the antioxidant system, endogenous phytohormones, and heat shock proteins	Khan et al., 2020b
*Triticum aestivum* L.	37 ± 2°C	2 and 4 mM Si	Si application increased the Chl a, b, and a + b and carotenoids by improving the activities of enzymatic antioxidants, CAT, SOD, POD, and osmoprotectants	Mustafa et al., 2021
*Triticum aestivum* L.	45°C, 4 h	1.5 mM K_2_SiO_3_ and 1.66 mM Si NPs	Si treatments restored the heat stress-provoked ultrastructural distortions of chloroplasts and the nucleus and enhanced photosynthetic capacity Si treatment also stimulated the overexpression of *TaPIP1* and *TaNIP2* with an improvement in the RWC	Younis et al., 2020
*Hordeum vulgare* L.	25, 30, and 35°C	1.5 mM Si	Si application reduced HT-mediated oxidative stress by decreasing the concentration of MDA (39 and 49%) and H_2_O_2_ (14 and 56%) and increased shoot (49 and 46%) and root (40 and 34%) dry masses, Chl a (10 and 86%), Chl b (82 and 81%), and carotenoids (53 and 33%)	Hussain et al., 2019
*Hordeum vulgare* L	34°C	0.2, 0.4, and 0.6 mM Na_2_O_3_Si.5H_2_O	Si treatment alleviated the detrimental impacts enhancing the antioxidant enzymes SOD, POD, CAT, and PPO, together with soluble sugars accumulation and free proline for osmotic adjustment	Naz et al., 2021b
*Euphorbia pulcherrima* L.	40°C	75 mg L^–1^ K_2_SiO_3_	Si treatment alleviates the temperature stress by regulating the stomata, photosynthesis, oxidative damage, and by lower production of H_2_O_2_ and MDA	Hu et al., 2020
*Zea mays* L.	12–14°C	40 mg H_4_SiO_4_ kg^–1^	Si treatment related to an improved Zn and Mn status maintains a balanced hormonal (IAA, GA, and CK) status that restores plant growth and helps to increase the expression of enzymatic (SOD and POD) and non-enzymatic (phenolic antioxidants) defense systems	Moradtalab et al., 2018
*Hordeum vulgare* L.	5°C and −5°C	56 mg L^–1^ as Na2SiO3	Si treatment increased the activity of antioxidative enzymes and concentrations of soluble carbohydrates and proteins	Joudmand and Hajiboland, 2019
*Aloe barbadensis* L.	4°C	Si 500, 1000, 1500, and 2000 mg L^–1^	Si treatment improved both enzymatic antioxidant activity and concentrations of soluble sugars	Azarfam et al., 2020
*Phyllostachys praecox*	5, 0, and −5°C	Si 0, 0.5, 1.0, 2.0, 4.0, or 8.0 g kg^–1^	Si treatment stimulated antioxidant systems and the enzyme activities of SOD, CAT, and POD Whereas the MDA content and cell membrane permeability decreased with all Si treatments	Qian et al., 2019

## Drought Tolerance Mediated by Silicon

The normal growth and the metabolism of plants are severely affected by drought stress, and, additionally, it creates a negative impact on ecosystem balance and agricultural systems (Kutasy et al., 2022). Drought markedly inhibits photosynthesis rather than respiration in crop plants (Vanlerberghe et al., 2016). Several studies approved the role of Si to mitigate the adverse effects of drought stress to crop plants and, hence, to regulate normal metabolism, such as photosynthesis and alleviation of photoinhibition (Habibi and Hajiboland, 2013; Zhu and Gong, 2014; Alamri et al., 2020; Ahmad et al., 2021; Malik et al., 2021). Some recent examples of Si-induced drought tolerance are presented in Table 2. The crop plants respond to temperature and drought stress by activating regulons, such as dehydration-responsive element-binding protein (*DREB2*) (Nakashima et al., 2012). For instance, exogenous supplementation of Si in tomatoes helps cope with drought to regulate osmotic adjustment and accumulation of drought-induced ROS (Cao et al., 2020a). Moreover, it is reported that drought-mediated accumulation of ROS can be circumvented through supplementation of Si to promote energy dissipation in tomato crop plants (Cao et al., 2020a). In a recent study, Kaya et al. (2006) have reported that Si highly enhanced the growth of shoots in maize under water-scarce conditions. In addition, drought stress can be mitigated by accumulating mineral nutrients like K^+^ ions induced by Si in wheat seedlings (Keller et al., 2015). Si results in enhanced uptake of macronutrient Ca, Mg, P, and K and micronutrients Cu, Mn, and Fe during drought stress in sunflower (Gunes et al., 2007). Water uptake was enhanced by Si-induced overexpression of *SbPIP1;6, SbPIP2;6*, and *SbPIP2;2*, encoding plasma membrane intrinsic protein (PIP) aquaporins in roots of *Sorghum bicolor* (Liu P. et al., 2015). Similar results were also displayed in rice where genes, such as *OsRDCP1*-encoding choline monooxygenase and dehydrin *OsRAB16b*, were upregulated under drought stress (Khattab et al., 2014). Several studies reported that Si supplementation enhanced root growth in crop plants under drought stress (Ahmed et al., 2011; Hameed et al., 2013).

Moreover, drought tolerance is incrementally enhanced by Si supplementation to alleviate the deficiency of minerals in plants (Habibi and Hajiboland, 2013; Bityutskii et al., 2017; Hosseini et al., 2017). Moreover, Verma et al. (2021) investigated that Si played a defensive role against drought stress in sugarcane. Another mechanism responsible for enhancing tolerance by Si depends on its capability to boost the binding of nutrients to plant tissues and also stimulates the translocation of nutrients from the root apoplast to the shoots (Greger et al., 2018). The versatility of Si to defend drought stress largely depends on its capability to induce the expression of genes to mitigate the stress. For instance, Kapoor et al. (2020) detailed the Si-regulated expression of genes coding for MYB, no apical meristem (NAC), C-repeat/dehydration-responsive element-binding factors (CBF/DREB), zinc-finger proteins (ZFPs), and cup-shaped cotyledon (CUC) to activate brought a tolerant-signaling cascade in plants. Additionally, Si also induces the expression of genes linked with flavonoid biosynthesis and elevation of genes pertaining to antioxidant enzymes to enhance drought tolerance in plants (Ma et al., 2016; Bhardwaj and Kapoor, 2021). Recently, under water-deficit conditions, the foliar application of Si has enhanced the antioxidants activities, osmolytes contents, growth, flowering, and yield of squash plants (Salim et al., 2021). These studies clearly indicate that Si is a central player in mitigating drought stress and further opens the wide scope to explore the molecular and genetic mechanisms to have in-depth knowledge for its resilience mechanism against drought.

## Heavy Metals Stress Tolerance Mediated by Silicon

The frequently found heavy metals, such as Al, Cd, arsenic (As), lead (Pb), mercury (Hg), copper (Cu), and zinc (Zn), in cultivated crop plants available through irrigation greatly influence the dynamics of soils (Shimo et al., 2011; Nazli et al., 2020; Asaad Bashir et al., 2021; Mehmood et al., 2021; Haseeb et al., 2022; Raza et al., 2022). These heavy metals induce devastating impacts on crop production and are potentially toxic to human health. The accumulation of heavy metals, such as Cd and Cu, binds to sulfhydryl groups of proteins, resulting in inhibition of major metabolic networks, such as lipid peroxidation, photosynthesis, and electron transport chain (ETC) (Sudo et al., 2008; Raza et al., 2021a,c). Crop production may be primarily affected by heavy metal ion-mediated adverse effects on tissues, such as induction of necrosis, leaf chlorosis, impairment in the synthesis of chlorophyll, and membrane lipid breakage (Sharma and Dietz, 2009; Takahashi et al., 2011; Raza et al., 2021d,^2022^). In the rhizosphere of crop plants, Si leads to exudates rich in chelated metal ions, hence reducing metal ion uptake (Khan et al., 2021a).

Additionally, due to the deposition of Si in the endodermis, a physical barrier is created to retard the uptake of metal ions through the xylem to reduce metal ion toxicity in wheat seedlings (Keller et al., 2015). The majority of crop plants like rice, barley, broad bean, summer squash, cucumber, and cowpea ameliorate Mn-based toxicity upon accumulation of Si (Liang et al., 2007). Si accumulators show considerably lower Mn toxicity, as they help reduce more than 90% of Mn in cell walls (Rogalla et al., 2002; Ahire et al., 2021; Mundada et al., 2021). The tolerance to Mn induced by Si is due to stronger affinity of Mn to cells walls of cucumber and cowpea (Iwasaki et al., 2002; Rogalla et al., 2002; Pavlovic et al., 2021). Moreover, Si is reported to enhance the tolerance of crop plants to salinity and heavy metals like Mn and Al (Liang et al., 2007). In addition, Si ameliorates the Cd toxicity in crop plants like wheat, cotton, rice, Bok choy, and peanut (Singh et al., 2019).

Additionally, Si mediates two critical functions in crop plants: reduced Cd uptake from shoots to grains and reduced uptake of Cd in plants (Fahad et al., 2015b). Moreover, it is documented that, in wheat plants, Si plays a vital role in Cd tolerance by augmenting the uptake of metals and inducing the translocation of metal-binding proteins associated with Cd (Greger et al., 2016). The concentration of Si ranging from 1.0–1.5 mM is reported to mitigate Cd toxicity in wheat, alfalfa, and rice. Liang et al. (2005) and Farooq et al. (2016) reported that tolerance to Cd in maize was obtained by administration of exogenous Si. Exogenous Si treatment in canola declined the adverse impacts of Cd on photosynthesis and other plant growth parameters (Baryla et al., 2001; Bukhari et al., 2021b). See Table 2 for some recent examples of Si-induced metal tolerance.

The possible mechanism of counteracting the toxic effects of Cd and Cu through the application of Si was correlated with overexpression of metal ion transporter genes, such as *OsLsi* and *OsHMA3* (Kim et al., 2014b). Moreover, Si resulted in declined Al and Cr (Chromium) uptake through roots, stems, and leaves of peanut and rice seedlings, ameliorating toxic effects of these heavy metals (Ali et al., 2013; Shen et al., 2014; Tripathi et al., 2015). Si in the form of silica gel helps to shuttle the influx transporter (Lsi1) to transport Si rather than arsenic, reducing its toxicity in rice (Rizwan et al., 2015). Comparative studies reported that Si supplementation enhanced the transcription levels of the *PsbY* (*Os08g02530*) gene-encoding polyprotein component of Photosystem II, whereas Zn administration downregulates its transcription in rice plants (Song et al., 2014). Under Zn stress, it is reported that Si mediates overexpression of the *PetC* gene, encoding Rieske Fe-S center-binding polypeptide of cytochrome *bf* complex, an essential component of cytochromes in plant electron chain system (Song et al., 2014). Under metal toxicity, it was reported that Si supplementation mediated positive effects on plants through enhancing chlorophyll biosynthesis and, in turn, photosynthetic machinery (Adrees et al., 2015; Imtiaz et al., 2016). The stomatal conductance, net photosynthetic quotient, transpiration ratio increase by enhanced synthesis of carotenoids, chlorophyll a, and b upon administration of Si under heavy metal stress in cotton and wheat plants (Bharwana et al., 2013; Hussain et al., 2015; Tripathi et al., 2015). Si supplementation downregulated the expression of zinc (Zn) transporters under zinc toxicity in maize roots (Bokor et al., 2015).

## Wound Stress Tolerance Mediated by Silicon

Plant wound stress occurs due to physical injuries usually induced by wind or herbivory. The damaged cells/tissue injuries are easily excess to bacterial or fungal origin pathogens. To be very specific, most of the time, injuries to C_3_ and C_4_ plants are primarily caused due to lodging (Savatin et al., 2014). Apart from injuries, unsaturated fatty acids of the plasma membrane are also damaged by wound-induced oxidative stress and lipid peroxidation (Farmer, 2005). Later response, i.e., lipid peroxidation, initiates a defensive signaling pathway mediated by the triggering of JA biosynthesis. Therefore, it is evident that wounding stress markedly results in increased lipid peroxidation and JA synthesis as reported in *A. thaliana* (Gfeller et al., 2011). Several reports have demonstrated the role of Si in providing strength to plant membranes and protection against injury during extreme stress conditions in crop plants, such as in *Paeonia lactiflora* Pall (Liang et al., 2007; Zhao et al., 2013). In response to oxidative stress, Si-supplied rice plants overexpress genes and activated oxidative enzymes, such as peroxidase (PO), catalase (CAT), and polyphenol oxidase (PPO), to alleviate the wound-induced oxidative damage (Kim et al., 2014b). Comparable trends were also observed in other crop plants, such as maize, wheat, and barley (Gong et al., 2005). In Si-administered crop plants, the silicified cells obstruct the pathogen entry at wound sites to impede the dissemination of infection (Ma et al., 2008). They may cause mandibular wear in grasshoppers due to abrasiveness in leaf tissue of rescue grass (Mir et al., 2019). The growth and the development of rice plants during the wound were maintained by exogenous supplementation of Si through enhancement of endogenous levels of ETH and JA.

## Temperature Stress Tolerance Mediated by Silicon

### Heat Stress

Crop production is deeply hindered by heat stress, resulting in the loss of economy and global food insecurity (Zhou et al., 2017; Raza, 2020). Furthermore, heat stress negatively affects tomato plant growth and production (Kumar and Kaushik, 2021; Ul Hassan et al., 2021). The rate of osmotic potential is impeached by heat stress, leading to an imbalance in water potential, resulting in the negative impact of biochemical pathways and tissue damage. To overcome the fluctuating temperatures, plants have evolved a diverse array of tolerance mechanisms, which include the production of heat shock proteins (HSPs), mechanisms to scavenge the ROS, and induction of specific phytohormones (Khan et al., 2020b; Haider et al., 2021a,b; Raza et al., 2021f). Khan et al. (2020b) reported that Si helps to induce thermotolerance by stimulating heat shock proteins, phytohormone production, and antioxidant system in tomatoes. Moreover, it is reported that small HSPs improve heat stress resistance in peanuts (Fragkostefanakis et al., 2016). Exogenous administration of Si was shown to enhance the tolerance to heat stress, resulting in improving shoot length and biomass production in sword fern, cucumber, and rice (Liu et al., 2009; Sivanesan et al., 2014). The heat tolerance in crop plants was inferred to Si-enhanced synthesis in photosynthetic pigments, such as chlorophyll a, chlorophyll b, and carotenoids in plants like grapevine and submerged macrophytes (Wang et al., 2010; Chalanika De Silva and Asaeda, 2017). The expression of *SlLsi1* and *SlLsi2* genes-encoding Si transporters was upregulated in Si-treated plants upon exposure to heat stress to improve heat tolerance in tomato plants (Khan et al., 2020b). See Table 2 for some recent examples of Si-induced heat stress tolerance.

Studies reveal that Si significantly triggered overexpression of heat tolerance genes, such as *HSFA3*, *HSFA5*, and *HSF30* in date palm and *DREB2, MAPK1, HSFA1a*, *HSFA1b*, *HSFA2*, *HSFA3*, and *HSFA7* in tomatoes (Khan et al., 2020b; Saha et al., 2021). For instance, Janni et al. (2020) reviewed the detailed mechanistic response of plants to overcome heat stress through activation of conserved pathways, viz. overexpression of ABA-responsive genes, Ca^2+^-sensing proteins, HSP-induced protein folding, and ROS-scavenging genes. Furthermore, it is reported that these signaling cascades lead to activation of *HSP* genes, such as *HSP18*, *HSP70*, and *HSP90*, through activation of HSFA2c to maintain folding and prevent non-specific protein formation under heat stress in tall fescue (Wang and Huang, 2017). Most remarkably, the activation of HSFs and HSPs interact with diverse signaling cascades triggered by Ca^2+^, phospholipids, SA, ET, ABA, H_2_O_2_, and NO to circumvent the negative impacts of heat stress on crop plants (Liu J. et al., 2015; Sharma et al., 2020). Hence, these reports strongly suggest that Si-induced HSPs are at the forefront of establishing proper folding, preventing denaturation and aggregation of cellular proteins, and inducing stay green traits through phytohormones under heat-stress conditions (Abdelrahman et al., 2017).

### Cold Stress

The cold stress, including the temperatures < 15°C referred to as chilling and below 0°C for freezing, drastically influences the development and growth of plants (Habibi, 2015). Cold stress damages lipids, proteins, carbohydrates, and nucleic acids through the production of ROS (Suzuki et al., 2012). To avoid this, plants have adopted several strategies to combat the cold stress for maintaining growth and development (Dey et al., 2022). Habibi (2015) reported that foliar-applied Si effectively alleviates the cold stress by maintaining the integrity of bio-membranes and decreasing photoinhibition in grapevine plants. Reports suggest that, upon Si treatment, plants modulate levels of phytohormones, such as JA, SA, and ABA, which, in turn, activate several cold tolerance-signaling pathways (Miura and Furumoto, 2013; Eremina et al., 2016; Rastogi et al., 2021). Moreover, Si helps to enhance the tolerance to cold stress by expressing a wide array of genes, most notably transcription factors. For instance, three cold-tolerant genes, viz. *DREB1A, DREB1B*, and *DREB1C*, transiently expressed during the cold stress in Arabidopsis to regulate growth (Liu et al., 1998; Lee et al., 2005; Gujjar et al., 2014). Subsequently, the overexpression of *OsDREB1* was reported to enhance tolerance to chilling and drought stress (Ito et al., 2006). Qian et al. (2019) reported that Si induced cold tolerance in *Phyllostachys praecox* by stimulating the activities of CAT, SOD, and POD during cold stress.

Similarly, Si enhances cold tolerance by affecting the hormonal balance and micronutrient homeostasis during early growth in maize plants (Bradáčová et al., 2016; Moradtalab et al., 2018). Earlier studies also reported that the Si-induced homeostasis of micronutrients is critical to protection under cold stress in maize (Imran et al., 2013; Bradáčová et al., 2016). Si alleviates the cold stress by modulating the concentration of metabolites and activity of apoplastic enzymes in leaf apoplasm in barley plants (Joudmand and Hajiboland, 2019). Recent reports have suggested that Si ameliorates the negative impacts of cold stress by regulating cellular redox homeostasis (Basu and Kumar, 2021; Mostofa et al., 2021). For instance, Hu et al. (2022) have recently reported that Si, in combination with selenium (Se), improves the yield, growth, and other physiological attributes of cucumber plants under field trails to circumvent extreme conditions, such as cold stress. See Table 2 for some recent examples of Si-induced cold stress tolerance. All these reports strongly suggest that Si plays a pivotal role in evading the negative impacts of cold stress on plants, even though scientists are still underway to explore the clear-cut molecular mechanism behind the resilience mediated by Si to alleviate cold stress.

## Oxidative Stress Tolerance Mediated by Silicon

The major consequence of environmental stress elevated by oxidative stress by augmenting ROS generation, which damages cellular proteins and biomembranes, hinders the host antioxidant system and impairs major metabolic activities (Bhardwaj and Kapoor, 2021). Against these reactive species, plants have evolved enzymatic as well as non-enzymatic antioxidant systems to protect against damage induced by oxidative stress (Hussain et al., 2016). For instance, Si-supplied plants withstand the harmful effects of ROS-induced oxidation of membrane lipids and other metabolic imbalances exposed to abiotic stresses, particularly heat stress (Chalanika De Silva and Asaeda, 2017; Li et al., 2018; Hussain et al., 2019; Merewitz and Liu, 2019). In a recent study, Abdelaal et al. (2020) have reported that sweet pepper Si alleviated the oxidative stress by accretion of protective proteins and decreasing the ion toxicity, hence enhancing the activity of oxidative enzymes and eliminating O^2–^ and H_2_O_2_ from cells. Si helps to enhance the activity of antioxidant enzymes, which, in turn, enhance the scavenging of ROS, declining lipid peroxidation (Liu P. et al., 2015; Coskun et al., 2019). Hasanuzzaman et al. (2020) reported that, even under optimal conditions, many metabolism processes produce ROS. Sorghum and sunflower were found to mitigate salt stress when Si was added to the growth medium (Liu P. et al., 2015; Yin et al., 2016; Hurtado et al., 2021). Foliar application of Si (150 mg L^–1^) in okra was found to enhance salt tolerance through its positive effect on activities of an antioxidant enzyme, leading to the reduction of lipid peroxidation (Abbas et al., 2015, 2017). Moreover, Si-alleviated salt stress in several crop plants like sorghum, sunflower, rice, wheat, maize, and mungbean, when supplemented in a growth medium (Liu P. et al., 2015; Abdel-Haliem et al., 2017; Alzahrani et al., 2018; Bosnic et al., 2018; Flam-Shepherd et al., 2018; Ahmad et al., 2019; Khan et al., 2020b; Hurtado et al., 2021). The Si supplemented in salt stress conditions resulted in increased activity of the antioxidant enzyme, leading to a decrease in the generation of ROS and reduced lipid peroxidation (Coskun et al., 2019). The lipid peroxidation and the production of H_2_O_2_ were alleviated by overexpression of the *OsNAC5* gene upon supplementation of Si (Hu et al., 2008). The ROS-scavenging enzymes, such as SOD, CAT, and GR, are upregulated upon exogenous administration of Si in barley to stand oxidative stress (Liang et al., 2003). In sorghum, wheat, rice, tomato, grapes, and okra, water uptake through aquaporins is improved by foliar application of Si due to reduced production of H_2_O_2_ (Soylemezoglu et al., 2009; Liu P. et al., 2015; Abbas et al., 2017; Abdel-Haliem et al., 2017). Overall, these reports validate the pivotal role played by Si to mitigate the oxidative stress by accretion of defense proteins, enhancing activity of antioxidant enzymes, reduction in membrane damage, and overexpression of ROS-scavenging genes.

## High-Throughput Approaches to Investigate Si-Induced Tolerance to Abiotic Stressors

Even though conventional breeding programs for combating abiotic stressors are achievable, the process is slow and tedious. The transfer of traits between species is incompatible and involves multiple backcrossing to screen desired traits out of thousands of genes (Ahmad and Mukhtar, 2017; Parmar et al., 2017). The traditional breeding methods are surmounted by genetic engineering techniques to precisely modify the gene of interest and its transfer to desired organisms (Singh and Singh, 2014). The genetic engineering approaches are aided by the advent of omics approaches to understand and identify genes/traits linked with the metabolic pathways critical to stress response, hence facilitating the transfer of identified traits to develop stress-tolerant crop plants (Mohanta et al., 2017).

High-throughput techniques, such as transcriptomics, proteomics, microarray analysis, and ionomics, have been widely used to identify differentially expressed genes or proteins in crop plants under control and stress conditions (Zargar et al., 2016; Khan et al., 2018; Yang and Guo, 2018; Afzal et al., 2022). The role of Si in alleviating stress is now obvious and has been reported to have a beneficial effect on plant growth and development under salt stress. However, the mechanism involved is not fully understood.

Transcriptomic studies are grossly employed to investigate the role of Si through the identification of a wide array of genes whose expression is altered in response to Si treatment under stress conditions. These genes are reportedly involved in solute transport, hormone biosynthesis and signaling, and biotic and abiotic stress response. A good percentage of differentially expressed genes in response to Si treatment are transcription factors (Zhu et al., 2019b; Arif et al., 2021). Liu P. et al. (2015) and Manivannan and Ahn (2017) reported that Si-upregulated *SbPIP* gene coding plasma membrane intrinsic protein (PIP) is vital for maintaining uptake of water by positively regulating the aquaporin activity and additionally enhanced salt tolerance in sorghum. Similarly, upregulation of stress-tolerant genes, such as *Os03g57120*, *Os09g26810*, and enhanced activity of S-adenosyl-L-methionine decarboxylase (SAMDC), to increase the synthesis of polyamines by Si is essential in mitigating the abiotic stressors (Manivannan and Ahn, 2017). The Si-upregulated expression of the *OsRDCP1* gene led to a decrease in dehydration of plant cells, hence mitigating drought stress (Khattab et al., 2014).

Transcriptome studies revealed that Si mitigates stress by upregulating the expression of genes *PsbY*, *PsaH*, *PsII*, and *LHC* involved in modulating PSII, oxygen-evolving manganese subunit PSI, and light harvesting complex activities during the photosynthesis process (Song et al., 2014; Lesharadevi et al., 2021). In wheat, Si improves heavy metal stress tolerance by increasing gene expression of metallothionein synthase 1 (*TaMT1*) and phytochelatin synthase (*TaPCS1*), and, in pea, Si application improves heavy metal stress tolerance by upregulating the expression of MTA (metallothionein) and GSH1 (a precursor of phytochelatin) genes in roots (Kaushik and Saini, 2019; Arif et al., 2021). Si upregulates the expression of transcription factors, such as dehydration-responsive element-binding protein (DREB2) and NAC, involved in mitigating osmotic stress *via* the ABA independent pathway (Manivannan and Ahn, 2017; Almutairi, 2019). Comparative transcriptome studies by Zhu et al. (2019b) suggested that Si acts as an elicitor to induce salt stress in cucumber. They reported that, among the genes whose expression was altered (708 upregulated and 774 downregulated) under salt stress, the majority of them (609 and 595, respectively) reverted to the normal expression levels upon Si treatment, shifting the transcriptome of salt-stressed cucumber back to normal.

The transcriptome analysis of different tissues, such as flag leaves (FLs), spikelets (SPs), and node Is (NIs) of Cd-exposed rice after Si application, revealed that the gene expression profiles associated with the Si-mediated alleviation of Cd stress were tissue-specific. Si may alter the expression pattern of genes associated with transport, biosynthesis and metabolism, and oxidation-reduction. In Cd-exposed rice, the expression of ATP-binding cassette (ABC)-transporters, essential nutrient transporters, carbohydrate, secondary metabolite biosynthesis, and cytochrome oxidase activity were mostly upregulated in FL and SP with Si treatment, while the expression of bivalent cation transporters was mostly downregulated, possibly to reduce Cd accumulation (Sun et al., 2022). In another study, Hao et al. (2021) used RNA-seq technology to identify DEGs in wheat seedlings treated with Si. They reported that 3,057 genes related to transcription factors, chaperons, phenylpropanoid biosynthesis, and protein processing were differentially expressed upon Si treatment. Approximately, 28 differentially expressed transcription factors from the MYB TF family were downregulated under Si treatment.

The proteomics offers a new approach to discovering a myriad of stress-inducible proteins that respond to abiotic stresses at transcriptional and translational levels to identify proteins and understand the pathways associated with stress tolerance (Kosová et al., 2011, 2018). Understanding the key metabolic proteins and pathways of stress tolerance can help in biotechnological interventions for the development of varieties with improved stress tolerance. Abiotic stresses interfere with protein synthesis and cause loss of protein. Si has been reported to efficiently lower stress by upregulating the synthesis of a protein associated with signal transduction processes and antioxidant defense machinery. Proteomics studies have identified that, upon exposure of plants to Si, 17% stress-tolerant proteins, 11% proteins related to cellular and hormone biosynthesis, RNA synthesis, and synthesis of secondary metabolites are upregulated to alleviate abiotic stressors (Al Murad et al., 2020). Likewise, proteomic studies have aided in tracing the upregulation of proteins, such as RNA polymerase mediator, homologs of transcription elongation (SPT4), MADS-box TF, TFs for elongation, tRNA-lysidine synthase, ribosomal protein L16, ribosome-recycling factor, and reverse transcriptase, by Si to confer tolerance against abiotic stress in plants (Muneer and Jeong, 2015; Al Murad et al., 2020).

Furthermore, proteomic studies have confirmed that Si mitigates environmental stressors, including salinity and thermal stresses by inducing synthesis of β-carotene synthesis protein carotene desaturase, chaperone protein, such as ClpC3, CF1α subunit of ATP-synthase, cupin family protein phosphoglycerate kinase, glutelin, and β-keto acyl reductase, plasma membrane intrinsic protein (PIP1), and nodulin 26-like intrinsic protein (NIP2) (Choi, 2009; Cao et al., 2020b; Younis et al., 2020). Like other grasses, rice accumulates Si and stores most of it in leaves. Study of change in leaf proteome in Si-induced Cd tolerance in rice has revealed that Si significantly regulated synthesis of proteins involved in photosynthesis, redox homeostasis, protein synthesis regulation, chaperone activity, and pathogen response, class III peroxidase, disulfide isomerase, an NADH-ubiquinone oxidoreductase alone, or in combination with Cd. It suggests a strong role of Si in induced tolerance (Nwugo and Huerta, 2011). A similar study on Si-induced salinity stress tolerance in *Capsicum annuum*, Manivannan et al. (2016) carried out proteomic analysis by two-dimensional gel electrophoresis (2DE), followed by matrix-assisted laser desorption/ionization time-of-flight mass spectrometry (MALDI-TOF-MS), revealed that Si treatment upregulated the accumulation of proteins with nucleotide-binding and transferase activity involved in several metabolic processes and also modulated the expression of vital proteins involved in the ubiquitin-mediated nucleosome pathway and carbohydrate metabolism (Manivannan et al., 2016).

Metabolomic studies helped to identify the accumulation of metabolites critical to the growth, sustenance, and development of plants under the influence of Si to mitigate adverse abiotic stress conditions (Jana et al., 2019; Raza, 2020). The Si upregulated the metabolites that include ascorbate, glutathione, phenol, tocopherol, and flavonoids to attenuate the harmful effects of abiotic stressors such that normal growth and metabolism will be sustained (Metwally et al., 2018). Recent ionomic studies have revealed that Si application induced the accumulation of critical cofactors of enzymes, such as Cu, Fe, and Zn, to mitigate stressors (Banerjee et al., 2021). Si application increases the accumulation of several nutrients, such as N, P, K, Mn, Fe, Na, Ca, and Mg, which reduce stress-induced ionic damage, increase drought tolerance, and enhance plant growth (Ahanger et al., 2020b; Pavlovic et al., 2021).

These omics reports serve as the backbone for applying genetic engineering approaches to mediate resilience in transgenic crop plants. For instance, the development of transgenic cultivars of rice plants through a transfer of the *Lsi1* gene modulated the physiological and molecular activities to mitigate arsenic stress upon exposure to Si (Boorboori et al., 2021). Likewise, overexpression of the *VyDOF8* transcription factor gene derived from Chinese wild grapevine was reported to mitigate drought stress in transgenic tobacco plants (Li et al., 2021). Similarly, *OsMADS57* TF was upregulated by Si, overexpressed in rice, and *A. thaliana* mitigated salinity stress. All these reports strongly suggest that genetic engineering technology speeds up the development of stress-resilient crop plants for food and nutritional security. The last few decades have witnessed shifting research on genome-editing technologies, such as ZFN, TALEN, and CRISPR/Cas systems, for higher accuracy and precision to develop desired crop plants with tolerance to different abiotic stressors (Mushtaq et al., 2021; Pandita, 2021). For instance, Li et al. (2019) employed CRISPR/Cas9 to mediate mutagenesis of *SlNPR1* to enhance drought tolerance in tomatoes. Almost no other report is available on the editing of the Si-encoding genes; thus, in the future, the CRISPR/Cas system should be applied to Si-encoding genes to increase the tolerance against various abiotic stresses in major crop plants. We believe the application of high-throughput technologies will aid in speeding up the quest to develop stress-tolerant crop varieties for food and nutritional security (Varshney et al., 2018, 2020, 2021a,b; Pazhamala et al., 2021).

## Conclusion and Future Perspectives

Even though the metabolic demand of Si is deficient, its presence in plant systems is critical to alleviating abiotic stress. Keeping in view the importance of Si to crop plants, the last decade witnessed many publications pertaining to its role in alleviating abiotic stress. Si is proven to play a vital role in combating abiotic stress conditions by positively modulating the physiological attributes of crop plants. Central to the role of Si is its upregulation of phytohormones and their signaling cascades operated to withstand abiotic stresses. Remarkably, Si is reported to impart more excellent resistance to abiotic stress factors through overexpression of genes regulating the synthesis of phytohormones. The stress tolerance mediated by Si-induced phytohormones is largely regulated through signal transduction pathways. Although the effect of Si to alleviate the abiotic stress in crop plants is variable, the multidimensional role of Si in plant development, such as the alleviation of sulfur deficiency, osmotic stress tolerance, alteration of genes related to glyceraldehydes-3-phosphate dehydrogenase, cyclophilin (CYC), ADP-Ribosylation Factor (ADP-RF1), and positively modulating metabolic pathways under abiotic stress, is well accustomed. The effect may vary concerning stress duration and intensity, *in vitro* methods of application, and cultivation. We conclude that Si can be integrated as a requisite part of fertilizers to benefit the crop plants in adverse ecological conditions. The focus on Si-based fertilizers may help to attain global sustainable agricultural growth.

The clear insights into the pivotal role of Si in circumventing the stress tolerance are still scanty, primarily due to a poor understanding of the molecular mechanism imparted by this multitalented element. Furthermore, extensive molecular investigation into the integrated effects of Si and phytohormones on alleviating the drought tolerance in plants is still at its early stage. In addition, the genes pertaining to the physiological improvements need to be further unveiled to authenticate the role of Si as the leading element in fertilizers for fortification and sustainable agricultural growth. Advanced omics technologies, such as transcriptomic, proteomic, and metabolomic studies, can further aid in-depth exploration of crosstalk of Si with phytohormones to unravel novel signaling molecules responsible for alleviating the stress tolerance in crop plants. In addition, special attention should be put on the role of Si in regulating signal transduction pathways under a wide range of abiotic stress conditions. Furthermore, shifting focus upon deciphering the role of Si in cop plants in felid trails rather than in controlled laboratory conditions is of dire need. In the future, the CRISPR/Cas system should be applied to Si-encoding genes to increase the tolerance against various abiotic stresses in major crop plants.

## Author Contributions

RM and SZ conceived the idea. RM, BB, HY, SI, MR, MA, PS, SC, and AR collected the literature and participated in writing. RM, BB, AR, SI, and SZ proofread and edited the final version. All authors have read and approved the final version of the manuscript.

## Conflict of Interest

The authors declare that the research was conducted in the absence of any commercial or financial relationships that could be construed as a potential conflict of interest.

## Publisher’s Note

All claims expressed in this article are solely those of the authors and do not necessarily represent those of their affiliated organizations, or those of the publisher, the editors and the reviewers. Any product that may be evaluated in this article, or claim that may be made by its manufacturer, is not guaranteed or endorsed by the publisher.
